# Supplementary material for: Efficacy of the Multi-Target Compound E153 in Relieving Pain and Pruritus of Different Origins
Source: Pharmaceuticals (Basel). 2023 Oct 17;16(10):1481. doi: 10.3390/ph16101481 (PMC10609854; doi:10.3390/ph16101481)
Supplement: Supplementary file 1 [file pharmaceuticals-16-01481-s001.zip › pharmaceuticals-2591879-supplementary.pdf]

Supplementary Material

Results

We aimed to test the influence of E153 on spontaneous locomotor activity to investigate its sedative properties. The high sedative activity of compounds is an undesirable property, which may lead to incorrect interpretation of the in vivo results and overinterpretation of the sedative effect as an analgesic or antipruritic effect.

The mean number of light-beam crossings in the vehicle-treated animals was 1.51 ± 0.13 x 103, measured during the 30 min-long period of observation. The test compound at a dose of 30 mg/kg significantly altered spontaneous locomotor activity in mice, but the lower dose of 15 mg/kg had no effect [$F(2,21) = 4.43$, $p < 0.05$] (Figure S1). Considering that the test compound significantly attenuated pain and pruritus at lower doses, we concluded that these effects did not result from its sedative properties.

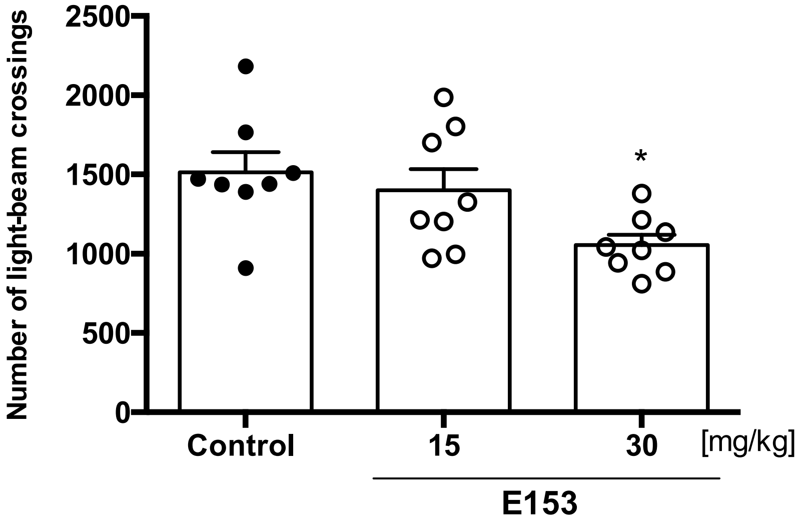

Figure S1. The influence of E153 on the spontaneous locomotor activity in mice. The test compound or vehicle (1% solution of Tween 80) were administered 30 min intraperitoneally (*i.p.*) before the beginning of the test. The results are presented as bar plots showing the mean ± SEM. Statistical analysis: one-way ANOVA followed by Dunnett's post hoc test, * $p < 0.05$, $n = 8$ mice per group.

The impairment of motor functions in the experimental animals induced by the test compound may subsequently cause a false interpretation of the results of the in vivo tests. To eliminate this risk, we assessed the influence of 153 on motor coordination using the rotarod test.

In this test, the vehicle-treated animals did not demonstrate impaired motor coordination. The time spent on the rotarod apparatus was 60 seconds for each control mouse. The same effect was observed in the E153- treated mice. Neither a dose of 15 mg/kg nor compound 30 mg/kg significantly impaired the motor coordination of mice at any tested speed [$F(2,15) = 0.41$, $p=0.67$] (Figure S2). We

concluded that the analgesic and antipruritic effects do not result from impaired motor function in E-153-treated animals.

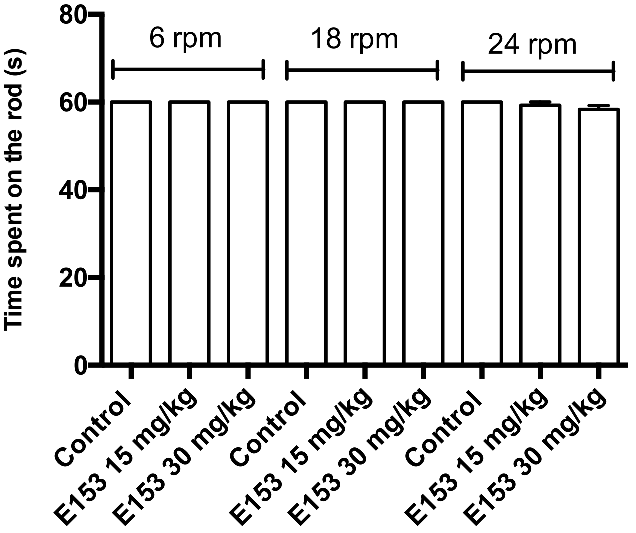

Figure S2. The influence of E153 on the motor coordination in the rotarod test. The test compound or vehicle (1% solution of Tween 80) were administered 30 min intraperitoneally (*i.p.*) before the beginning of the test. The results are presented as bar plots showing the mean ± SEM. Statistical analysis: one-way ANOVA followed by Dunnett's post hoc test, n = 6 mice per group.

Methods

The methodes were briefly described by Mogilski et al. [1].

References

1. Mogilski; Kubacka, M.; Łażewska, D.; Więcek, M.; Głuch-Lutwin, M.; Tyszka-Czochara, M.; Bukowska-Strakova, K.; Filipek, B.; Kieć-Kononowicz, K. Aryl-1,3,5-triazine ligands of histamine H4 receptor attenuate inflammatory and nociceptive response to carrageen, zymosan and lipopolysaccharide. *Inflamm. res.* **2017**, *66*, 79–95.
